# An unexpected high-pressure stability domain for a lower density polymorph of benzophenone

**DOI:** 10.1038/s41598-023-38985-y

**Published:** 2023-07-24

**Authors:** I. B. Rietveld, M. Barrio, R. Ceolin, J. Ll. Tamarit

**Affiliations:** 1 SMS Laboratory (UR 3233, Université Rouen-Normandie, Place Émile Blondel, 76821 Mont Saint Aignan, France; 2 Faculté de Pharmacie, Université Paris Cité, 4 Avenue de l’observatoire, 75006 Paris, France; 3 Grup de Caracterització de Materials, Departament de Física and Barcelona Research Centre in Multiscale Science and Engineering Universitat Politècnica de Catalunya, EEBE, Campus Diagonal-Besòs, Av. Eduard Maristany 10-14, 08019 Barcelona, Catalonia Spain

**Keywords:** Chemical physics, Thermodynamics

## Abstract

For over a century, it was thought that the crystalline polymorph II of benzophenone does not possess a stable domain in the pressure–temperature phase diagram. With a combination of new experimental results and literature data, this case of crystalline dimorphism has finally been solved and it is shown that form II possesses a stable domain at high pressure and high temperature, even though its density is lower than that of form I, the stable form under ordinary pressure and temperature conditions. The phase diagram of benzophenone is a clear demonstration of the fact that to understand the phase behaviour of a chemical substance both the exchange of heat (due to the change in intermolecular interactions) and work (due to the change of volume at a given pressure) need to be taken into account.

## Introduction

Phase theory was established by Josiah Willard Gibbs in the nineteenth century based on earlier work by Europeans such as Clapeyron and Clausius^1–5^. His theory was quickly adopted and used to understand phase behaviour by researchers such as Bakhuis-Roozeboom^6^. The essence of the work by Gibbs is that each phase has a different (Gibbs) energy (Gibbs himself used the internal energy U) and that stability is found in the phase with the lowest energy by exchanging heat and work with the surroundings. For crystalline phases and written in terms of the change in internal energy dU, work is exchanged as − PdV, thus in the form of a volume change against an external pressure and heat is exchanged as TdS, thus an increase in entropy (“release of heat”) against a given temperature. In terms of a change in the Gibbs energy dG, these terms become VdP and -SdT, so for the Gibbs energy, the variables are pressure and temperature:

1 $${\text{dG }} = \, - {\text{SdT }} + {\text{ VdP}}$$

The actual value of the Gibbs energy for a given phase will be determined by the integration over P and over T, but it can be seen from Eq. (1) that a polymorph with a smaller volume will contribute less to the Gibbs energy at high pressure, than a polymorph with a larger volume. Thus, with an increase in pressure, the denser polymorph becomes more stable. In a similar vein, a crystal with a higher entropic content contributes more to a lower Gibbs free energy, as this term is negative. Entropy is a measure of the heat content and translates within a crystal into the strength of the intermolecular interactions and into the vibrational freedom of the molecules. The intermolecular interactions and vibrational freedom are obviously linked, but they also represent two distinct contributions to the entropic content in terms of the overall bond enthalpy and of the vibrational energy. In a liquid, intermolecular interactions are relatively weak while simultaneously molecules have ample possibilities to vibrate, so the entropic content is high, whereas in a strongly bound solid, with strong hydrogen bonds for example, the entropic content is low due to the “concentrated” energy involved in the strong hydrogen bonding and the lower vibrational freedom. As will become clear in the rest of the article, the dimorphism of benzophenone is an excellent example of the necessity to take both heat (the entropic term, − SdT) and work (the volume term, VdP) into account in the evaluation of the overall Gibbs free energy of a polymorph.

For more than a hundred years, benzophenone is known to exhibit crystalline dimorphism; however, it has always been thought that the metastable form was inherently metastable as a literature review demonstrates. In a historical overview of the polymorphism of benzophenone (C_13_H_10_O, M = 182.217 g mol^−1^), Kutzke et al. reported that no conditions are known for which the monoclinic form II (referred to as β form) is more stable than the orthorhombic form I (α form)^7^. Tonkov mentioned in his compilation of benzophenone phase equilibria under pressure that Bridgman on increasing the pressure isothermally at 25 °C did not observe any phase transition up to 2.5 GPa^8^. Muller, in 1914, had previously classified the metastable form II among those forms he named “totally unstable forms”^9^ based upon the inequality in melting temperatures between the two polymorphs, as well as inequalities in enthalpy and volume changes on melting. More precisely, one can read in the abstract of Muller’s paper that “since it has been found that* T*_fus,I_ > *T*_fus,II_, ∆*v*_I→L_ > ∆*v*_II→L_ *and* ∆_fus_*H*_I_ > ∆_fus_*H*_II_, it is improbable that the two forms can exist in equilibrium with one another at a transition point. It is probable, therefore, that the unstable form is totally unstable. This is confirmed by the direction of the fusion curves of the two forms, which has been calculated by the equation d*T*/d*p* = *T*·∆_fus_*v*/∆_fus_*H*. The values obtained show that the direction of the curve for the stable form deviates but slightly from that for the unstable form…. From a study of the condition diagram [i.e. the pressure–temperature phase diagram], it is highly improbable that the stable and unstable forms can exist together in equilibrium at high pressures”^9^. The same argumentation was developed by Tammann who mentioned the case of the less stable form of benzophenone when discussing “on the melting curves of unstable forms”^10^. Later, Bridgman dealing with “melting phenomena under pressure”, used the same arguments and he mentions that a polymorph can only become more stable with increasing pressure if it possesses a higher density, thus in principle excluding stability domains at high pressure for less dense polymorphs. It has to be added that Bridgman referred here to isothermal increase of pressure, a point that is rather important as will be seen further on in this paper^11^.

An example of the systems described by Bridgman is paracetamol, which has a low-density polymorph that is stable under ordinary conditions (without applied pressure). A denser form, that crystallises in certain cases, only possesses a stable pressure–temperature domain at high pressure. Although a topological phase diagram was published by Espeau et al. in^12^ and several structural studies have been carried out^13–15^, experimental evidence for the stability domain of the metastable phase of paracetamol at high pressure was provided by Ledru et al. in^16^. Other systems that properly obey the inversion of stability from a less dense to a denser phase on increasing the pressure are for example the legendary ritonavir^17–20^ and the more mundane progesterone^21^. Another example, that is similar to the behaviour of paracetamol is resorcinol polymorphs α and β^22^. Here again the more stable form at high pressure is the high density form β, but in this case a high temperature transition from α and β before fusion, excluded in paracetamol^16^, is observed too; however, the precise layout of the phase diagram still needs to be investigated.

Coming back to the benzophenone system, the historic studies and in particular the analysis of Muller^9^, point in the direction that form II possesses no stable domain at all in the pressure–temperature phase diagram. That would mean that the system would be overall monotropic with form I the only stable solid form irrespective of the pressure, which is the fourth case of dimorphism described by Bakhuis–Roozeboom^6,23^. This would place the phase behaviour of benzophenone among that of drug molecules such as biclotymol and rimonabant^24,25^ and that of simpler molecules such as monochloroacetic acid and hydrazine monohydrate^26^. Since the specific volume of form II and as a result inequalities between the two forms have become available thanks to the work of Kutzke et al.^7^, thermodynamic data from the literature, together with new measurements, were examined to investigate whether a topological pressure–temperature phase diagram for this historical case of dimorphism could be constructed from data at ordinary pressure using the Clapeyron equation. In addition, the pressure dependence of the melting temperature of form II has been obtained for the first time by high-pressure differential thermal analysis, and its slope was compared to the topologically determined slope.

## Literature data

### Specific volume and thermal expansion of the two polymorphs

The Cambridge Structural Database (CSD) contains crystal structures of the two known polymorphs of benzophenone. Specific volumes from the CSD data have been compiled in the Supplementary Material Table S1^7,27–33^. They have been graphically represented in the Supplementary Material Fig. S1, and a linear fit of the specific volumes to the temperature gave the following result:

2 $$v_{\text{I}} /{\text{cm}}^{3}{\text{g}}^{ - 1} = \, 0.774\left( 4 \right) \, + \, 0.00016\left( 2 \right)T/{\text{K}}\;\;\;\; \left( {r^{2} = \, 0.90} \right)$$

The three specific volumes of form II obtained from the structures determined by Kutzke et al.^7^ and by Bernstein et al.^33^ lead to the following dependence on the temperature for the specific volume of form II:

3 $$v_{{{\text{II}}}} /{\text{cm}}^{{3}}{\text{g}}^{{ - {1}}} = \, 0.{781}\left( {8} \right) \, + \, 0.000{15}\left( {3} \right)T/{\text{K}} \left( {{\text{r}}^{{2}} = \, 0.{92}} \right)$$

The straight lines represented by these two equations are very close and the values at room temperature are almost undistinguishable (cf. Fig. S1) even if the value found by Bernstein^33^ suggests that the specific volume of form II may be larger than that of form I.

Thus, from the analysis of the literature on the difference in the specific volumes between the two forms, it appears that:

1. the volumes are very close, and the thermal expansion leads to practically parallel lines *v* = f(*T*),
2. the specific volume of form II, *v*_II_, seems to be slightly larger than *v*_I_,
3. the two forms exhibit virtually the same expansivity of about 2 × 10^–4^ K^−1^ (see Fig. S1 in the Supplementary Materials).

Therefore, new measurements, reported below in the results section, were carried out on a single powder X-ray diffractometer to analyse more precisely the behaviour of the respective specific volumes.

### Specific volume and thermal expansion of liquid benzophenone

Values of the specific volume of liquid benzophenone found in the literature have been compiled in Table S2 of the Supplementary Material^34–37^. Comparison of these data demonstrates that the results of a very recent investigation by Kerscher et al.^36^ using a DMA 4200 Anton Paar vibrating densitometer are very close to the much older results by Jaeger^35^. Nonetheless, for the highest consistency only the recent results have been used to derive an equation for the thermal expansion of the liquid state:

4 $$v_{{\text{L}}} /{\text{cm}}^{{3}}{\text{g}}^{{ - {1}}} = \, 0.{7}0{14}\left( {9} \right) \, + \, 0.000{683}\left( {3} \right)T/{\text{K}} \;\;\;\;\; \left( {{\text{r}}^{{2}} = \, 0.{9998}} \right)$$

### Temperatures and heats of fusion

Available temperature and heat of fusion data of benzophenone forms I and II have been compiled in Table S3 in the Supplementary Material^9,35,38–46^. The mean values are: *T*_fus,I_ = 321.3(3) K, *T*_fus,II_ = 298.7(8) K, ∆_fus_*H*_I_ = 18.0(8) kJ mol^−1^ (99(5) J g^−1^), and ∆_fus_*H*_II_ = 14.0(5) kJ mol^−1^ (77(3) J g^−1^).

### Heats of sublimation and of vaporization

For form I and the liquid, literature data of respectively sublimation and vaporization have been compiled in Table S4 in the Supplementary Material in the form of the Clausius–Clapeyron equation^44,46–54^:

5 $$\mathrm{ln}p /\mathrm{Pa}=A-\left(\frac{B}{T/\mathrm{K}}\right)$$

in which *A* is a constant and *B* is the ratio of the vaporization or sublimation enthalpy and the gas constant *R*. The enthalpy can be derived from B as has been done in Table S4 in the Supplementary Materials. The average value of the sublimation enthalpy for form I equals ∆_sub_*H*_I_ = 92.8(2.9) kJ mol^−1^ (509(16) J g^−1^) and the average value for the vaporisation enthalpy of the liquid (only two values) equals 73.5(3) kJ mol^−1^ (403.6(1.3) J g^−1^). The difference between form I and the liquid is fusion implying that the difference between the latter two enthalpies should be equal to the enthalpy of fusion of form I. This leads to ∆_fus_*H*_I_ = 105(17) J g^−1^, which is somewhat larger than the mean value of the direct measurements reported in the literature in Table S3 of 99(5) J g^−1^; however, within the standard deviations of the data the values are the same.

Recently, Stejfa et al.^46^ reported extensively on the thermodynamic data of the phase changes of benzophenone to revise and improve its thermodynamic data. They measured the temperatures and heats of fusion of forms I and II, together with the vapor pressures of the condensed phases to which sublimation and vaporization enthalpies could be fitted [see Supplementary Material Tables S3 and S4 and Eqs. (S1)–(S4)]. Their data and the resulting equations allowed the calculation of the transition enthalpies at specific temperatures: ∆_sub_*H*_I_(*T*_fus,I_ = 321.3 K) = 94.27 kJ mol^−1^ (517.3 J g^−1^), ∆_vap_*H*(*T*_fus,I_ = 321.3 K) = 75.77 kJ mol^−1^ (415.8 J g^−1^), ∆_sub_*H*_II_(*T*_fus,II_ = 298.3 K) = 92.35 kJ mol^−1^ (506.8 J g^−1^), and ∆_vap_*H*(*T*_fus,II_ = 298.3 K) = 77.78 kJ mol^−1^ (426.9 J g^−1^) using Eqs. (S2), (S3), and (S4), respectively^46^. This would lead to an enthalpy of fusion for form I of 101.5 J g^−1^ and for form II of 80.0 J g^−1^, very close to the values obtained by DSC. These authors observed a glass transition at *T*_g_ = 212 K^46^, which is the same value as found by Davydova et al.^55^ and independently by Romanini et al.^56^.

### Solid–liquid equilibrium of form I as a function of pressure

The solid–liquid equilibrium of form I as a function of the pressure has been widely studied in a large pressure range up to 3 GPa by a number of authors^34,38,57–60^. A compilation of the available data from the literature can be found in Table S5 in the Supplementary Material. All of the different data sets, with the exception of Akella and Kennedy’s results^60^, can be described by a linear equation leading to slopes of the melting equilibrium in the pressure–temperature phase diagram, d*p*/d*T*_I→L_, ranging from about 3.5 to 4.7 MPa K^−1^. The average value of the slopes of the five data series, which could be fitted with a straight line, equals 3.97(52) MPa K^−1^.

## Results

### Calorimetric data at ordinary pressure

DSC measurements carried out in this study led to melting enthalpies (Table 1) that are somewhat larger than those compiled in Table S3 in the Supplementary Material. They are, however, close to those reported by Stejfa et all. (See Table S3, row marked with reference 22 of the Supplementary Materials)^46^. It should be stressed that, in all cases, ∆_fus_*H*_I_ was found to be larger than ∆_fus_*H*_II_.

**Table 1:** Temperatures and heats of fusion of forms I and II obtained by DSC.

	*T*_fus,I_/K	∆_fus_*H*_I_/J g^−1^	*T*_fus,II_/K	∆_fus_*H*_II_/J g^−1^
	321.55	108.93	298.30	81.31
	321.13	109.18	298.19	86.66
	321.21	110.72	298.36	80.12
	320.71	97.71	298.26	85.67
	320.74	106.44	298.28	85.94
	320.83	104.84	298.13	80.42
	320.53	107.54		
	320.71	109.96		
	321.11	102.70		
	320.61	101.00		
Mean^a^	320.9 (4)	106 (5)	298.25 (9)	83 (3)

^a^Standard deviation between parentheses.

### Lattice parameters and specific volumes of benzophenone forms I and II as a function of temperature

**Figure 1:**
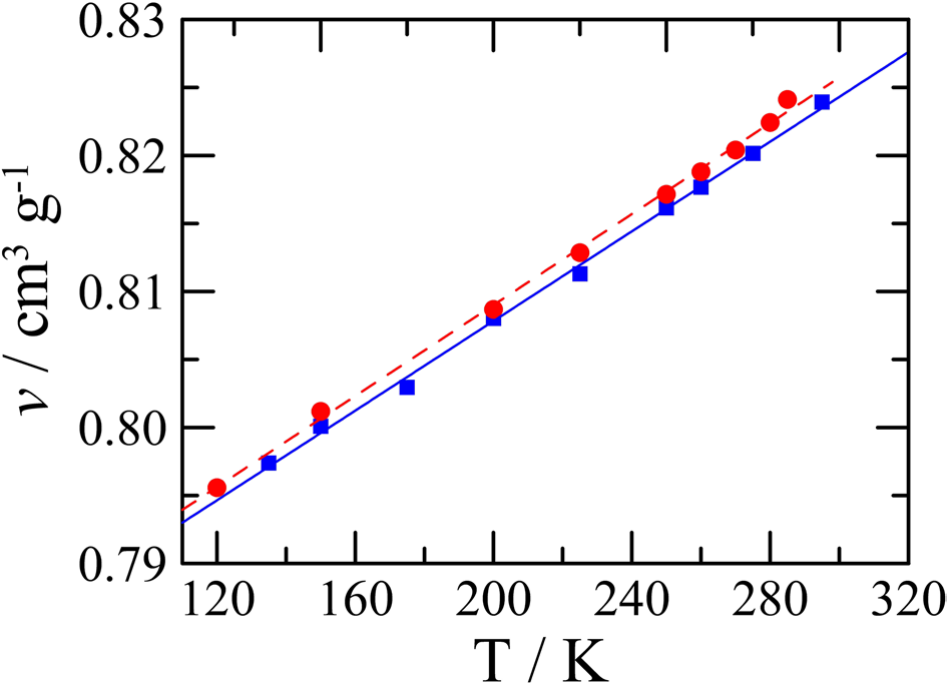
Specific volumes of benzophenone form I (blue filled square) and form II (red filled circle) as a function of temperature, while the straight lines are the fitted equations: blue is Eq. (6) and red Eq. (7).

The literature data have already demonstrated that the difference in specific volume between the two forms is quite small, while the specific volume of form I appears to be somewhat smaller. To determine the inequality in the specific volumes of forms I and II as precisely as possible, diffraction experiments on the two samples were carried out with the same number of data acquisitions, the same acquisition times and within the same temperature range. The specific volumes resulting from the diffraction experiments can be found in Fig. 1, while the refinement data have been compiled in the Supplementary Material in Table S6. These data lead to the following linear equations for the specific volumes of the two forms:

6 $$v_{{\text{I}}} /{\text{cm}}^{{3}}{\text{g}}^{{ - {1}}} = \, 0.{7749}\left( {7} \right) \, + \, 0.000{165}\left( {3} \right)T/{\text{K}} \;\;\;\; \left( {{\text{r}}^{{2}} = \, 0.{998}} \right)$$

7 $$v_{{{\text{II}}}} /{\text{cm}}^{{3}}{\text{g}}^{{ - {1}}} = \, 0.{7756}\left( {7} \right) \, + \, 0.000{167}\left( {3} \right)T/{\text{K}}\;\;\;\; \left( {{\text{r}}^{{2}} = \, 0.{998}} \right)$$

These results are comparable to the literature data in which form II possesses a slightly larger specific volume than form I; hence, the latter is the denser form. The expansivities of the two forms can be calculated from the Eqs. (6) and (7). They are α_v,I_ = 2.13 × 10^–4^ K^−1^ from Eq. (6) for form I and α_v,II_ = 2.16 × 10^–4^ K^−1^ from Eq. (7) for form II.

### Melting equilibria of forms I and II as a function of pressure

**Figure 2:**
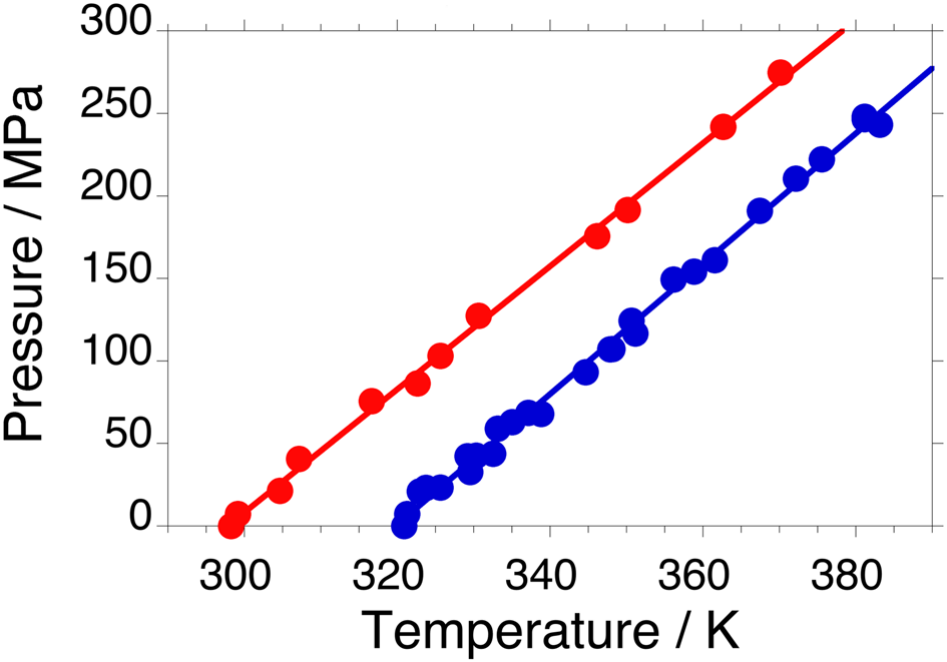
Pressure–temperature melting equilibria of benzophenone form I (blue filled circle) and form II (red filled circle), while the lines are the respective fits [Eqs. (8) and (9)].

With the DTA endotherms recorded at various pressures up to about 250 MPa (some peaks are presented in Figs. S2a and b in the supplementary material), the onsets of the melting temperatures of forms I and II have been determined and can be found in Fig. 2 and in Table S7 in the Supplementary Material. The data were fitted to the following equations:

8 $$P_{{{\text{I}} \to {\text{L}}}} /{\text{MPa }} = { 3}.{95}\left( {5} \right)T/{\text{K}} - {1263}\left( {{18}} \right) \;\;\;\; \left( {{\text{r}}^{2} = \, 0.{996}} \right)$$

9 $$P_{{{\text{II}} \to {\text{L}}}} /{\text{MPa }} = { 3}.{73}\left( {6} \right)T/{\text{K}} - {111}0\left( {{19}} \right) \;\;\;\; \left( {{\text{r}}^{{2}} = \, 0.{998}} \right)$$

The slope of the I–L equilibrium obtained in this study, 3.95(5) MPa K^−1^, is equal within error to the average of the literature values of 3.97 MPa K^−1^.

## Discussion

### The I–II-L triple point

For the first time, the pressure–temperature relationship of the melting of form II has been obtained by direct measurement. It demonstrates that the II-L equilibrium intersects the I-L equilibrium at high pressure, because the slope of the former is less steep, d*P*/d*T*_II-L_ = 3.73(6) MPa K^−1^, than the slope of the latter, d*P*/d*T*_I-L_ = 3.95(5) MPa K^−1^, while the II-L equilibrium is located at a higher pressure around room temperature. To calculate the coordinates of the intersection, which represents the I-II-L triple point, where form I, form II and the liquid are in equilibrium, Eqs. (8) and (9) are set equal. This leads to a triple point temperature of 692 K and, from either Eqs. (8) or (9), one can find the triple point pressure to be 1470 MPa. Thus, with the current slopes in Eqs. (8) and (9), the two equilibria slowly converge with increasing pressure and temperature.

The convergence of the two equilibria with increasing temperature and pressure is corroborated by the topological method that has been described in Methods as will be shown in the following. Volume changes on melting are assessed and the d*P*/d*T* slopes of the melting equilibria are calculated using Eq. (12). In addition, the equation of the elusive I-II equilibrium curve is calculated, and the topological P–T phase diagram of the benzophenone dimorphism is constructed and compared with the experimental one.

### Volume changes on melting

The specific volumes of the two solids have been determined in this study resulting in Eqs. (6) and (7) for form I and form II, respectively. The specific volume of liquid benzophenone can be calculated with Eq. (4) obtained with data from the literature. For the change of volume on melting of form I, the specific volumes need to be evaluated at the melting temperature *T*_fus,I_ = 320.9 K. For form I, this results in 0.82775 cm^3^ g^−1^ and for the liquid in 0.92043 cm^3^ g^−1^, which leads to ∆*v*_I→L_ = 0.09267 cm^3^ g^−1^.

With Eqs. (4) and (7), the specific volume of the liquid at the melting point of form II at *T*_fus_,_II_ = 298.3 K can be determined to be 0.90496 cm^3^ g^−1^ and for form II one finds 0.82544 cm^3^ g^−1^. This leads to a volume change on melting of ∆*v*_II→L_ = 0.07952 cm^3^ g^−1^.

### The slopes and equations of the melting equilibria in the P–T diagram

To calculate the slope of an equilibrium in the pressure–temperature phase diagram, the Clapeyron equation [Eq. (12)] needs the enthalpy change (Table 1), the equilibrium temperature (Table 1), and the volume change [Eqs. (4), (6) and (7)] between the two phases in equilibrium. This leads to the following slopes for the two melting equilibria: d*p*/d*T*_I-L_ = 3.56 MPa K^−1^ and d*p*/d*T*_II-L_ = 3.51 MPa K^−1^.

As can be seen in Fig. 2, the dependence of the melting temperature on the pressure for these two equilibria are straight lines at least up to 250 MPa. The intersection with the pressure axis at a temperature of 0 K, can be found with the understanding that the equilibrium pressure of the melting solid (or its liquid) can be set equal to 0 MPa at the temperature axis under ordinary conditions in a DSC pan. This is because the liquid is not boiling, and its pressure must therefore be smaller than 1 bar or 0.1 MPa. One obtains therefore:

10 $$P_{{{\text{fus}},{\text{I}}}} /{\text{MPa }} = { 3}.{56}T/{\text{K }}{-}{ 1143}$$

11 $${\text{P}}_{{{\text{fus}},{\text{II}}}} /{\text{MPa }} = { 3}.{51}T/{\text{K }}{-}{ 1}0{48}$$

The coordinates of triple point I-II-L can once again be found by setting equal Eqs. (10) and (11), resulting in: *T*_I–II-L_ = 2030 K and *P*_I–II-L_ = 6084 MPa.

These values are higher than the ones obtained through the measured pressure–temperature curves; however, they corroborate that the intersection of the two equilibria occurs at high pressure and temperature.

### The elusive I–II equilibrium

The transition from one form to the other one has never been experimentally observed. However, applying the Le Chatelier principle, it can be inferred under which conditions and in which direction it may occur:

1. Because Hess’ law leads to the conclusion that the transition from form I to form II is endothermic, ∆_I→II_*H* = ∆_fus_*H*_I_ – ∆_fus_*H*_II_ = 22.5 J g^−1^, the I–II equilibrium will shift towards form II on heating.
2. Because the specific volume of form II is slightly larger than that of form I (see Fig. 1), the I–II equilibrium will shift towards form I on increasing the pressure.

Moreover, the temperature of the I–II equilibrium at “ordinary pressure”, calculated with the formula proposed by Yu^61^ and the mean values in Table 1, is found to be 446 K. This is clearly larger than the melting temperature of form I at 321 K. Using Eqs. (6) and (7), the difference in the specific volumes between the two solid phases at the calculated transition temperature *T*_I→II_ = 446 K can be calculated. This leads to a value of *v*_II_–*v*_I_ = 0.0018 cm^3^ g^−1^ in the case of an endothermic shift from form I to form II. Using this value in the Clapeyron equation with the transition temperature *T*_I→II_ = 446 K and the enthalpy difference ∆_I→II_*H* = 22.5 J g^−1^ leads to a steep, positive slope in the pressure–temperature phase diagram of d*p*/d*T*_I–II_ of about 28 MPa K^−1^. Considering that this slope is much steeper than the two melting equilibria, either directly measured or calculated with the Clapeyron equation, it confirms the finding that the I-II-L triple point must be located at high temperature and high pressure. Any other set of data from the literature leads to similar conclusions and demonstrates a convergence of the melting equilibria and the solid–solid equilibrium at high pressure and temperature.

### Topological pressure–temperature phase diagram of the dimorphism of benzophenone

**Figure 3:**
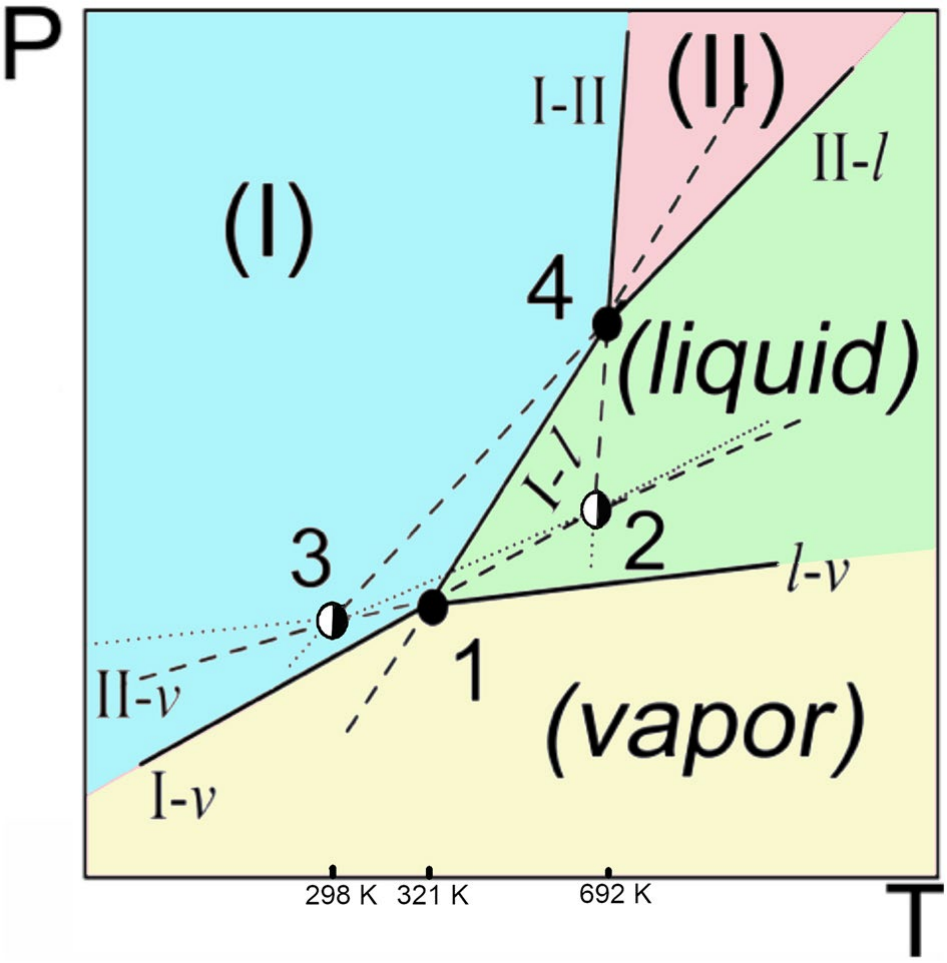
Topological pressure–temperature phase diagram for the dimorphism of benzophenone. Sky blue domain: stable phase region of form I, Red domain: stable phase region of form II. Black filled circle: stable triple points with 1: I–L-vapor and 4: I–II-L, black filled semi-circle: metastable triple points with 2: I–II-vapor and 3: II-L-vapor. Solid lines: stable two-phase equilibria, dashed lines: metastable two-phase equilibria, dotted lines: supermetastable two-phase equilibria. The temperature and pressure axes are not to scale.

Results inferred from the Clapeyron equation confirm the experimental high-pressure data of the melting equilibria of the two benzophenone polymorphs: they converge on increasing the pressure. The slope of the I–II equilibrium is positive and much larger than those of both melting curves. Because these three curves intersect at triple point I–II-L, the topological pressure–temperature phase diagram for this case of dimorphism should be drawn as shown in Fig. 3. In this figure, it is assumed that the melting points at “ordinary pressure” approximate the triple points involving the vapor phase. This type of phase diagram is an example of the third case of the four possible cases of dimorphism formerly proposed by Bakhuis Roozeboom^6,23^, in which a monotropic system turns enantiotropic under pressure. Examples of such systems are paracetamol^16^, progesterone^21^, and ritonavir^19^; however, the particularity of the benzophenone system, which it shares with metacetamol^62^, bicalutamide^63^, and fluoxetine nitrate^64^, is that a lower density phase only possesses a stable domain under pressure and at high temperature.

The whole reason why these low-density polymorphs can become stable at higher pressures, whereas under atmospheric conditions, only the densest form is stable, is due to the fact that both heat and work affect the Gibbs energy of a phase. The volume is obviously affected by the pressure, and a lower volume is favoured by a higher pressure; however, if at the same time the temperature is increased, the entropic contribution of the phase, its “heat”, also needs to be taken into account according to Eq. (1). Although this may appear logical, it is often overlooked as the introduction on the study of the phase behaviour of benzophenone clearly demonstrates and there is also very little data on how often this type of phase behaviour occurs.

An example that is worth additional study is bis-3-nitrophenyl disulphide, which was shown to crystallise under pressure and under increased temperature into low density polymorphs^65^. In the paper, these polymorphs have been presented as kinetic polymorphs^65^, which may imply that they are formed out of equilibrium. It still needs to be established whether these polymorphs are actually stable under the conditions at which they crystallise, or whether they indeed are purely kinetic polymorphs following Ostwald’s rule of stages. In the former case, they will in fact add to the examples of high-pressure, high-temperature, low-density, stable polymorphs.

## Concluding remarks

The current system reinforces the existing statistical data on thermal expansion and volume change on melting. From Eqs. (4), (6), and (7), it can be concluded that the expansivities for form I, form II and the liquid phase are respectively *α*_v,I_ = 2.13 × 10^–4^ K^−1^, *α*_v,II_ = 2.16 × 10^–4^ K^−1^, and *α*_v,L_ = 9.73 × 10^–4^ K^−1^. These values virtually match the mean values reported by Gavezzotti^66^. Moreover, the ratio *v*_L_/*v*_solid_ at the melting equilibrium for form I equals 1.11 with a volume change of 0.09267 cm^3^ g^−1^, while for form II 1.10 is found with a volume change of 0.07952 cm^3^ g^−1^. These values are very similar or equal to 1.11 proposed by Goodman et al.^67^ and Barrio et al.^68^ Furthermore, at *T*_g_ = 212 K, the specific volume of the metastable liquid is 0.8461 cm^3^ g^−1^, while the stable form I possesses a specific volume of 0.8098 cm^3^ g^−1^, which leads to a *v*_L_/*v*_I_ ratio at *T*_g_ of 1.05. Once more, this falls within the proposed range of ratios between 1.04 and 1.07 for the ratio between the liquid and the stable solid at the glass transition temperature^68^.

Although for over a century, form II of benzophenone was considered a fully metastable phase, the present paper demonstrates that form II does possess a stable domain in the pressure–temperature phase diagram at high temperature and high pressure. This has been demonstrated by using available data in the literature and by new measurements, which all point to the stable domain at high pressure. In particular, the slope of the melting equilibrium of form II as a function of pressure, which has been measured for the first time, provides a strong argument for the stable domain at high pressure.

It can be concluded from the phase diagram that it is not possible to obtain form II by isothermally increasing the pressure on form I, because this will only stabilize form I. Although, it may be possible to increase the pressure and then the temperature to obtain a transition from form I to form II, a more convenient way is to obtain form II by increasing the pressure on the liquid as this will force recrystallisation into form II once the temperature and pressure surpass the triple point. The fact that form II was thought to be fully metastable is, however, not extremely surprising in the sense that it is not immediately evident why a less dense form would possess a stability domain at high pressure. Thus, as stated in the introduction and in the discussion, thermodynamic stability should always be evaluated by taking into account the changes in both heat and work.

## Methods

### Obtention of forms I and II

A commercial batch of benzophenone form I from Across, purity = 99%, was further purified by sublimation-condensation under a vacuum, which allowed suitable single crystals to be grown.

To obtain a powder of form II for X-ray diffraction measurements as a function of temperature, part of the purified crystals was molten and maintained at 373 K for one hour. A Lindemann capillary was immersed in the liquid, which was drawn into the capillary. The capillary was cooled in situ in the diffractometer (see below) below the temperature of the glass transition and then reheated until recrystallization into form II was observed. The capillary was subsequently cooled once more for the X-ray diffraction acquisition as a function of temperature.

Samples of form II for the high-pressure measurements were obtained in the following manner. Crystals of form I were melted inside the measurement pan followed by an increase in pressure up to at least 150 MPa, at which it was maintained while cooling to room temperature. On heating, various starting pressures were set to obtain the melting temperature of form II as a function of the pressure.

### High-resolution powder X-ray diffraction experiments as a function of temperature

PXRD measurements have been carried out with a vertically mounted INEL cylindrical position-sensitive detector (CPS-120) using the Debye–Scherrer geometry and transmission mode. Monochromatic Cu-Kα_1_ (λ = 1.54056 Å) radiation was selected by means of a focusing incident-beam germanium monochromator. Measurements as a function of temperature were carried out using a liquid nitrogen 700 series Cryostream Cooler from Oxford Cryosystems. Cubic Na_2_Ca_3_Al_2_F_4_ was used for external calibration. The PEAKOC application from DIFFRACTINEL software was used for the calibration as well as for the peak position determinations after pseudo-Voigt fittings and lattice parameters were refined by way of the least-squares option of the FullProf suite^69,70^.

Benzophenone samples were introduced in a Lindemann capillary (0.5-mm diameter) as described above in the crystal obtention section. The capillaries were rotated perpendicularly to the X-ray beam during the experiments to improve the averaging over the crystallite orientations. Before each isothermal data acquisition, the samples were allowed to equilibrate for about 10 min, and each acquisition time was no less than 1 h. The heating rate in-between data collection was 1.33 K min^−1^. Patterns were recorded on heating in the temperature range from 100 K up to the melting point.

### Differential scanning calorimetry

DSC experiments have been carried out using a Q100 thermal analyser (from TA Instruments, USA) on heating at a 10 K min^−1^ rate with initial form I and form II recrystallized from the glass. The analyzer was calibrated with the melting point of indium (*T*_fus_ = 429.75 K and Δ_fus_*H* = 3.267 kJ mol^−1^). The specimens were weighed using a microbalance sensitive to 0.01 mg and sealed in aluminum pans.

### Temperature dependence of the fusion of forms I and II on pressure

High-Pressure differential thermal analysis (HP-DTA) measurements have been carried out at 2 K min^−1^ using an in-house constructed high-pressure differential thermal analyser similar to Würflinger’s apparatus and operating in the temperature and pressure ranges of respectively 298–473 K and 0–250 MPa^71^. To determine the melting temperature as a function of pressure and to ascertain that in-pan volumes were free from residual air, specimens were mixed with an inert perfluorinated liquid (Galden®, from Bioblock Scientifics, Illkirch, France) as a pressure-transmitting medium, and the mixtures were sealed into cylindrical tin pans. To verify that the perfluorinated liquid was chemically inactive and would have no influence on the melting temperature of benzophenone, preliminary DSC measurements were carried out with a Galden®-benzophenone mixture on a Q100 analyser of TA instruments without applied pressure.

### Methodology for the construction of a topological pressure–temperature phase diagram

The Clapeyron equation provides the initial value of the slope of a two-phase equilibrium in a pressure–temperature phase diagram of a one-component system:

12 $$\frac{dp}{dT}=\frac{\Delta S}{\Delta V}=\frac{\Delta H}{T\Delta V}$$

with ∆*S* the entropy difference between the two phases at equilibrium and ∆*V* their specific volume difference under the same conditions. For experimental reasons, ∆S is generally replaced by ∆*H*/*T*, because the enthalpy difference, ∆*H*, can be directly obtained by DSC at the equilibrium temperature, *T*, under ordinary pressure and at the equilibrium temperature ∆*S* = ∆H/*T*, because ∆*G* = 0. Ordinary pressure is the saturated vapor pressure of the system itself, often (but not always) negligible.

The phase equilibrium curves in a pressure–temperature phase diagram are projections of the intersections of monotonic Gibbs energy, *G*(*p*,*T*), surfaces. These equilibrium curves are therefore also monotonic, and they can often be drawn as straight lines over an extensive pressure range^72,73^. Each pair of phase equilibria intersects only once at so-called triple points. The triple points are the points of intersection of three two-phase equilibria whose stability ranking shifts on intersection at the triple point. Moreover, due to the necessary consistency in the stability ranking around a triple point, the phase equilibria are alternatingly stable and metastable (or they possess a lower stability hierarchy; however, the difference in ranking around a triple point is always of one single level i.e., stable-metastable, metastable-supermetastable, etc.).

The change in volume at equilibrium is obtained from the projection of the Helmholz function, *A*(*V*,*T*), on a volume-temperature plane^74^. The experimental thermal expansions of the condensed phases in the system are assumed to be obtained under saturated vapor pressures of the system.

## Supplementary Information

Supplementary Information.

## Data Availability

All data generated or analysed during this study are included in this published article (and its supplementary information files).
