# Supplementary material for: An unexpected high-pressure stability domain for a lower density polymorph of benzophenone
Source: Sci Rep. 2023 Jul 24;13:11914. doi: 10.1038/s41598-023-38985-y (PMC10366203; doi:10.1038/s41598-023-38985-y)
Supplement: Supplementary file 1 — Supplementary Information. [file 41598_2023_38985_MOESM1_ESM.pdf]

SUPPLEMENTARY INFORMATION FOR:
AN UNEXPECTED HIGH-PRESSURE STABILITY DOMAIN FOR A LOWER DENSITY POLYMORPH OF BENZOPHENONE

By I.B. Rietveld, M. Barrio, R. Ceolin, J.-Ll. Tamarit

Table S1. Specific volumes of the two polymorphs of benzophenone available from the literature

CSD acronym	Temperature /K	Vcell /Å^3	Specific volume /$cm^3 \cdot g^{-1}$	Reference
**Form I (or α), orthorhombic, space group $P2_12_12_1$, Z = 4**				
**BPHENO01**	283-303	977.462	0.80759	[1]
**BPHENO10**	283-303	1001.16	0.82717	[2]
**BPHENO12**	90	954.146	0.78833	[3]
**BPHENO13**	300 (neutrons)	992.159	0.81967	[4]
**BPHENO14**	70 (neutrons)	949.164	0.78431	[4]
**BPHENO15**	293	1001.706	0.82781	[4]
**BPHENO17**	293	999.08	0.82545	[5]
**BPHENO18**[a]	100<br>123	957.67(12)[c]<br>959.71(5)	0.79124(10)[c]<br>0.79292	[6]
**BPHENO19**[b]	100<br>123	957.57(34)[d]<br>960.27(5)	0.79116(30)[d]<br>0.79339	[6]
**Form II (or β), monoclinic, space group C2/c, Z = 8**				
**BPHENO02**	285	1988.351	0.82140	[7]
**BPHENO03**	293<br>223	1996.536<br>1966.152	0.82478<br>0.81223	[7]
**BPHENO11**	232	1976.871	0.81666	[8]

[a] (MM)-benzophenone
[b] (PP)-benzophenone
[c] mean of 4 values
[d] mean of 3 values

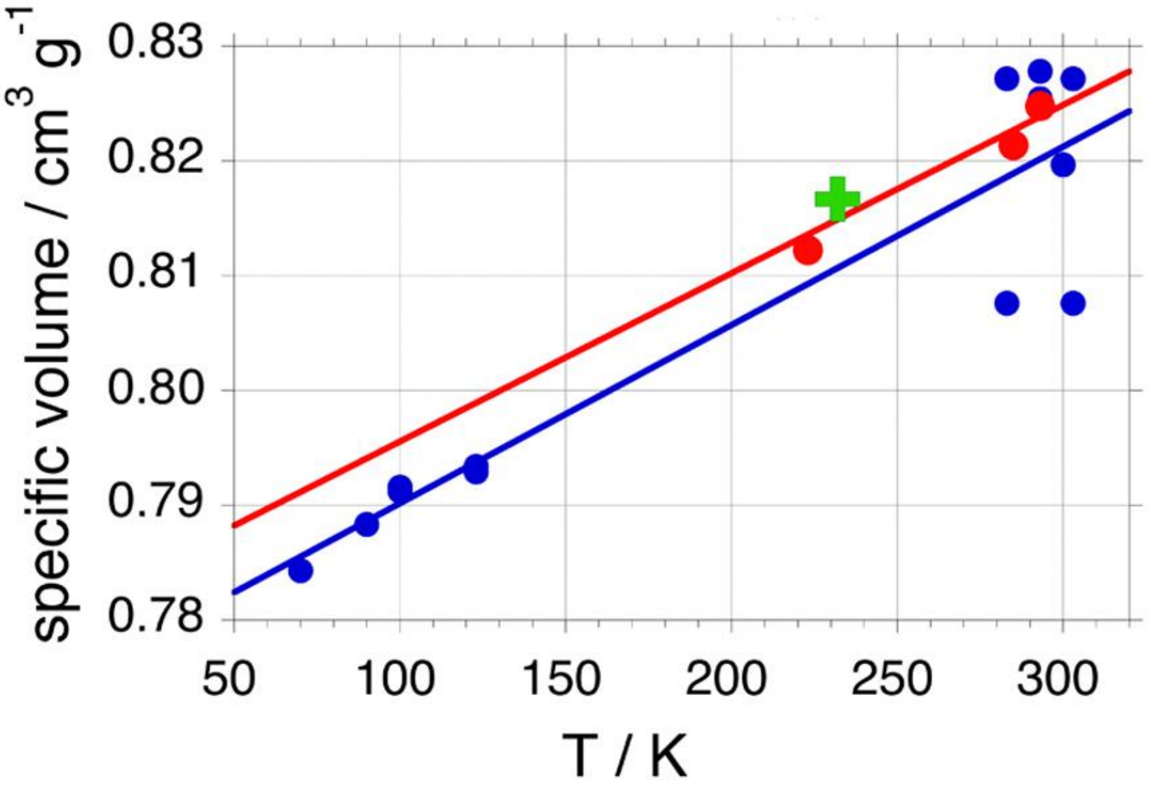

Figure S1. Specific volumes of benzophenone forms I and II as a function of temperature from the literature (see Table S1). Blue circles: form I, red circles: form II[7], green cross: form II[8], blue line: main text **eq. 1**, red line: **eq. 2**. Expansivities: $\alpha_{v,I} = 2.09 \times 10^{-4}$ K^{-1} from **eq. 1**, $\alpha_{v,II} = 1.88 \times 10^{-4}$ K^{-1} from **eq. 2**.

Table S2. Data from the literature for the specific volume of liquid benzophenone as a function of the temperature

Temperature /K	v_L /cm^3·g^{-1}	Reference	Temperature /K	v_L /cm^3·g^{-1}	Reference
**298.15**	0.89783	9	**340.07**	0.93990	10
**308.15**	0.90551	9	**343.47**	0.94360	10
**321.25**	0.91704	9	**323.45**	0.91996	11
**323.15**	0.91814	9	**338.15**	0.93023	11
**313.08**	0.92060	10	**348.15**	0.93721	11
**314.57**	0.92170	10	**364.15**	0.94787	11
**318.47**	0.92470	10	**377.25**	0.96246	11
**321.27**	0.92680	10	**394.15**	0.97276	11
**324.67**	0.92850	10	**403.65**	0.97943	11
**327.48**	0.92960	10	**424.15**	0.99701	11
**330.57**	0.93370	10	**444.95**	1.0152	11
**333.37**	0.93580	10	**457.45**	1.0277	11
**337.27**	0.93860	10	**473.15**	1.0417	11
**283.15**	0.89511	12	**323.15**	0.92167	12
**288.15**	0.89835	12	**328.15**	0.92509	12
**293.14**	0.90162	12	**333.15**	0.92854	12
**298.14**	0.90491	12	**338.15**	0.93201	12
**303.15**	0.90822	12	**343.15**	0.93550	12
**308.15**	0.91155	12	**348.15**	0.93902	12
**313.15**	0.91490	12	**353.15**	0.94257	12
**318.15**	0.91827	12	**358.15**	0.94614	12
			**363.15**	0.94974	12

Table S3. Literature values of the temperature and heat of fusion of benzophenone forms I and II[a]

Form I		Form II		Reference
$T_{fus,I}$ /K	$\Delta_{fus}H_I$ /kJ·mol^{-1}(J·g^{-1})	$T_{fus,II}$ /K	$\Delta_{fus}H_{II}$ /kJ·mol^{-1} (J·g^{-1})	
**321.15**	17.955 (98.54)			13
**321.2**	17.67 (96.97)			14
**321.1**	17.84 (97.91)	**298**	13.65 (74.91)	15
**321.65**	-	**299.65**	-	11
**-**	16.54 (90.77)			16
**321**	17.82 (97.80)	**299**	13.81 (75.79)	17
**321.35**	16.90 (92.75)			18
**322**	19.30 (105.92)			19
**321.03**	18.194 (99.85)			20
**321.28**	18.47 (101.36)			21
**321.1[b]**	18.606 (102.11)[b]			13,b
**321.3**	18.6 (102.08)	**298.3**	14.5 (79.58)	22
**321.3(3)**	18.0(8) (99(5))	**298.7(8)**	14.0(5) (77(3))	Mean

[a] If necessary, reported values in degree Celsius and in calory have been converted following the equalities: 0°C = 273.15 K and 1 cal = 4.184 J

[b] Bridgman[13] had compiled "older" T_{fus} and $\Delta_{fus}H_I$ values ranging from 47.7 to 48.5°C (i.e. 320.8 – 321.5 K) and from 23.2 to 23.9 cal·g^{-1} (i.e. 17.69-18.22 kJ·mol^{-1}), respectively; the values listed in this row are the averages of those reported values.

Table S4. Literature data of the sublimation and vaporization pressure of benzophenone forms I, II and the liquid in the form of coefficients A and B[a,b]

A	B	ΔH /kJ·mol^{-1}	Reference
Form I			
**33.767**	10818	87.97	23
**29.431**	10819.5	89.96	24
**35.96**	11426	95.00	25
**35.915**	11434	95.07	20
**34.842**	11119	92.45	26
**35.398**	11433	95.06	27
**-**	-	94.39/95.02/ 94.22	28
**-**	-	91.21/94.98/94.56/ 93.35/92.05/89.96	29
**42.009**	11167	92.85	29
**-**	-	84.39	30
**35.959**	11445	95.16	22
Form II			
**35.509**	11156	92.76	22
Liquid			
**27.815**	8825.7	73.38	20
**27.945**	8865.6	73.71	27

[a] See main text **eq. 4**

[b] ΔH: the enthalpy of sublimation for the two solids and the enthalpy of vaporization for the liquid

The vapour pressure (p_{vap} /Pa) of the liquid as a function of the temperature (T /K) obtained by Stejfa et al.[22] gave rise to a quadratic function, not listed in **Table S4** above:

$$\ln p_{vap} = 21.77 - 4682.2\ T - 7.0725 \times 10^5/T^2 \qquad (r^2 = 0.99999) \qquad (S1)$$

The dependence on the temperature (T /K) of the enthalpy differences (ΔH /kJ mol^{-1}; '$\Delta_{vap}H$' for the liquid, '$\Delta_{sub}H$' for the solids) between the condensed phases and the vapour was also reported by Stejfa et al.[22] It led to the following equations:

$\Delta_{vap}H = 104.46 - 0.089303\,T$ ($r^2 = 0.9991$) (S2),

$\Delta_{sub}H_{I} = 103.79 - 0.029643\,T$ ($r^2 = 0.9995$) (S3),

$\Delta_{vap}H_{II} = 102.41 - 0.033673\,T$ ($r^2 = 0.999$) (S4).

Table S5. Literature data for the pressure of fusion of form I as a function of temperature

**G.A. Hulett[31]**		**G. Tammann[32]**		**P.W. Bridgman[13]**	
**T/K**	P/MPa	T/K	P/MPa	T/K	P/MPa
**321.95**	2.5331	320.85	0.0000	320.92	0.098067
**322.70**	5.0663	328.11	29.126	347.75	98.066
**323.45**	7.5994	328.11	25.988	372.05	196.13
**324.15**	10.133	338.46	64.430	394.45	294.20
**324.60**	12.666	338.46	63.645	415.15	392.27
**325.50**	15.199	348.19	103.07	434.45	490.33
**326.30**	17.732	348.19	101.89	452.75	588.40
**327.10**	20.265			470.15	686.47
**327.75**	22.798			486.85	784.53
**328.45**	25.331				
**329.20**	27.864				
**329.90**	30.398				
**L. Deffet[33]**		**H. Block[10]**		**J. Akela et al.[34]**	
**T/K**	P/MPa	T/K	P/MPa	T/K	P/MPa
**321.15**	0.0000	321.11	0.098067	321.65	0.0000
**324.15**	10.003	334.60	49.033	435.15	500
**328.15**	24.124	346.89	98.066	505.15	1000
**333.15**	43.149	359.17	147.10	563.15	1500
**338.15**	60.801	370.81	196.13	618.15	2000
**343.15**	79.826	381.30	245.17	673.15	2500
**347.15**	94.340	389.14	294.20	725.15	3000
**348.15**	98.066				

Linear fits derived from the data in **Table S5**:

Hulett:[31] $P = 3.4921\,T - 1121.7$ ($r^2 = 0.999$) (S5),
Tammann:[32] $P = 3.7244\,T - 1195.1$ ($r^2 = 0.999$) (S6),
Bridgman:[13] $P = 4.7452\,T - 1555.6$ ($r^2 = 0.994$) (S7),
Deffet:[33] $P = 3.6534\,T - 1174.1$ ($r^2 = 0.9999$) (S8),
Block:[10] $P = 4.2485\,T - 1372$ ($r^2 = 0.995$) (S9).

Table S6a. Lattice parameters and specific volumes of benzophenone form I, orthorhombic unit cell, as a function of temperature[a]

T /K	$a(\sigma)$ /Å	$b(\sigma)$ /Å	$c(\sigma)$ /Å	$V_{cell}(\sigma)$ /Å^3	$v(\sigma)$ /cm^3·g^{-1}
**135**	12.071(1)	10.254(1)	7.7974(3)	965.1(2)	0.79740(11)
**150**	12.079(11)	10.262(11)	7.8124(6)	968.4(2)	0.80009(13)
**175**	12.083(1)	10.262(1)	7.8377(4)	971.8(2)	0.80295(11)
**200**	12.100(1)	10.273(1)	7.8678(5)	978.0(2)	0.80803(12)
**225**	12.106(1)	10.273(1)	7.8957(4)	981.9(2)	0.81130(12)
**250**	12.117(1)	10.281(1)	7.9295(6)	987.8(2)	0.81615(13)
**260**	12.123(1)	10.285(1)	7.9373(4)	989.7(2)	0.81767(12)
**275**	12.127(1)	10.289(1)	7.9556(5)	992.7(2)	0.82015(12)
**295**	12.133(1)	10.291(1)	7.9868(4)	997.2(2)	0.82393(12)

[a] Standard deviation (σ) in parentheses

Table S6b. Lattice parameters and specific volumes of benzophenone form II, monoclinic unit cell, as a function of temperature[a]

T /K	$a(\sigma)$ /Å	$b(\sigma)$ /Å	$c(\sigma)$ /Å	$\beta(\sigma)$ /°	$V_{cell}(\sigma)$ /Å^3	$v(\sigma)$ /cm^2·g^{-1}
**120**	16.164(1)	8.0250(3)	16.096(1)	112.724(3)	1925.8(2)	0.79558(8)
**150**	16.181(1)	8.0507(4)	16.145(1)	112.759(4)	1939.4(2)	0.80119(9)
**200**	16.200(1)	8.0841(4)	16.213(1)	112.786(4)	1957.6(2)	0.80869(9)
**225**	16.217(1)	8.1006(4)	16.247(1)	112.794(4)	1967.6(2)	0.81285(9)
**250**	16.234(1)	8.1189(4)	16.282(1)	112.820(4)	1978.0(2)	0.81714(9)
**260**	16.238(1)	8.1264(4)	16.297(1)	112.828(4)	1982.1(3)	0.81880(9)
**270**	16.240(1)	8.1351(4)	16.312(1)	112.850(4)	1985.9(3)	0.82040(9)
**280**	16.241(1)	8.1464(4)	16.333(1)	112.887(4)	1990.8(3)	0.82242(9)
**285**	16.247(1)	8.1545(4)	16.347(1)	112.908(4)	1994.9(3)	0.82412(9)

[a] Standard deviation (σ) in parentheses

Table S7. Melting pressure as a function of the temperature for forms I and II of benzophenone

T /K	$P_{I\to L}$ /MPa	T /K	$P_{II\to L}$ /MPa
**320.91**	0.0	**298.25**	0.0
**321.32**	7.4	**299.15**	7.3
**322.95**	21.0	**304.65**	21.3
**323.75**	23.3	**307.15**	40.7
**325.65**	23.3	**316.65**	75.8
**329.18**	42.4	**322.65**	86.5
**329.55**	32.7	**325.65**	103.1
**330.35**	42.7	**330.65**	127.4
**332.55**	43.9	**346.15**	175.7
**333.15**	59.1	**350.15**	191.5
**335.05**	63.0	**362.65**	242.0
**337.15**	68.5	**370.15**	274.8
**338.85**	67.9		
**344.65**	93.3		
**347.85**	107.1		
**348.15**	107.2		
**350.65**	124.5		
**351.15**	116.7		
**356.15**	149.4		
**358.85**	154.3		
**361.55**	161.1		
**367.45**	191.0		
**372.15**	210.5		
**375.55**	222.4		
**381.15**	248.0		
**381.15**	246.6		
**383.15**	243.2		

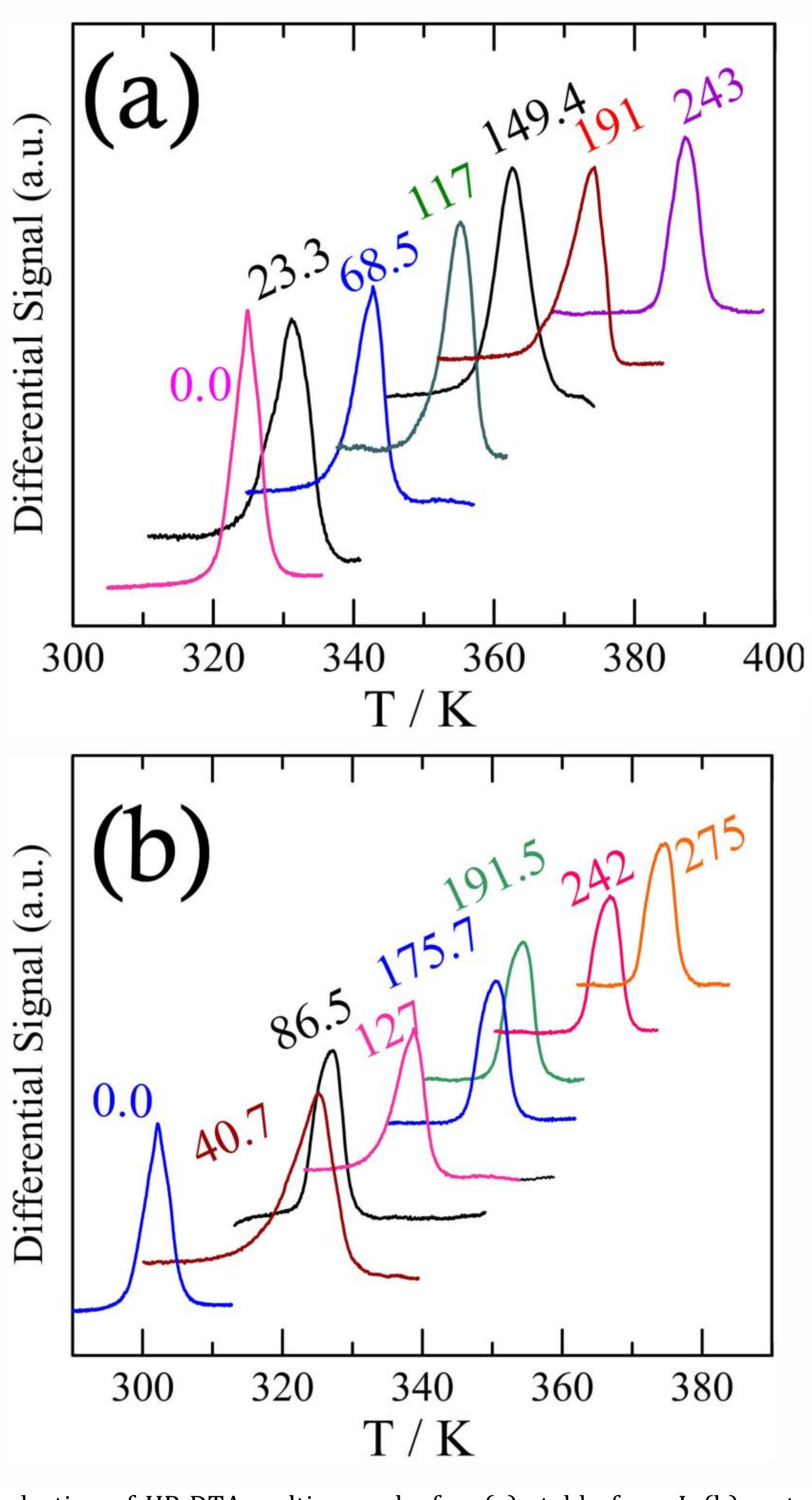

Figure S2. A selection of HP-DTA melting peaks for: (a) stable form I, (b) metastable form II. Pressure values in MPa reported with the same colours as the melting peaks.

References in the Supplementary Materials

1 Lobanova, G. M. Molecular and crystal structure of benzophenone. *Kristallografiya* **13**, 984 (1968).

2 Fleischer, E. B., Sung, N. & Hawkinson, S. Crystal structure of benzophenone. *J. Phys. Chem.* **72**, 4311, doi:10.1021/j100858a065 (1968).
3 Moncol, J. & Coppens, P. in *Benzophenone; private communication to CSD* (CCDC, 2004).
4 Reilly, A. M. *et al.* Predicting anisotropic displacement parameters using molecular dynamics: density functional theory plus dispersion modeling of thermal motion in benzophenone. *J. Appl. Crystallogr.* **46**, 656-662, doi:10.1107/s0021889813006225 (2013).
5 Yadav, H., Sinha, N., Tyagi, N. & Kumar, B. Enhancement of Optical, Piezoelectric, and Mechanical Properties in Crystal Violet Dye-Doped Benzophenone Crystals Grown by Czochralski Technique. *Cryst. Growth Des.* **15**, 4908-4917, doi:10.1021/acs.cgd.5b00792 (2015).
6 Matsumoto, A. *et al.* Absolute structure determination of chiral crystals consisting of achiral benzophenone with single-crystal X-ray diffraction and its correlation with solid-state circular dichroism. *Chem. Lett.* **45**, 526-528, doi:10.1246/cl.160114 (2016).
7 Kutzke, H., Klapper, H., Hammond, R. B. & Roberts, K. J. Metastable β-phase of benzophenone: independent structure determinations via X-ray powder -diffraction and single crystal studies. *Acta Crystallogr. B* **56**, 486-496, doi:10.1107/s0108768100000355 (2000).
8 Bernstein, J., Ellem, A. & Henck, J. O. in *Benzophenone; private communication to CSD* (CCDC, 2002).
9 Tarantino, D. E., Kohn, J. P. & Brenneke, J. F. Phase equilibrium behaviour of the carbon dioxide + benzophenone binary system. *Journal of Chemical & Engineering Data* **39**, 158-160 (1994).
10 Block, H. Über die Volumenänderung beim Schmelzen von Kristallen und die Wärmeausdehnung der Kristalle und ihrer Schmelzen. *Z.Phys.Chem.(Leipzig)* **78**, 385-425 (1911).
11 Jaeger, F. M. Über die Temperaturabhängigkeit der molekularen freien Oberflächenenergie von Flüssigkeiten im Temperturbereich von -80 bis +1650 °C. *Zeitschrift fur Anorganische und Allgemeine Chemie* **101**, 1-214 (1917).
12 Kerscher, M. *et al.* Viscosity, surface tension, and density of binary mixtures of the liquid organic hydrogen carrier diphenylmethane with benzophenone. *Int. J. Hydrogen Energy* **47**, 15789-15806 (2022).
13 Bridgman, P. W. Changes of phase under pressure. II. New melting curves, with a general thermodynamic discussion of melting. *Phys. Rev.* **6**, 94-112, doi:10.1103/physrev.6.94 (1915).
14 Eykmann, J. F. Zur kryoskopischen Molekulargewichtsbestimmung. *Z.Phys.Chem.(Leipzig)* **4**, 497-519 (1889).
15 Muller, A. H. R. Über total instabile Formen. *Z.Phys.Chem.(Leipzig)* **86**, 177-242 (1914).
16 Straton, K. & Partington, J. R. Latent heats of fusion. I. Benzophenone, phenol, and sulfur. *Philos. Mag. (1798-1977)* **43**, 436 (1922).
17 Timmermans, J. *Les constantes physiques des composés organiques cristallisés*. 465 (Masson, 1953).
18 Rastogi, R. P., Nigam, R. K., Sharma, R. N. & Girdhar, H. L. Entropy of fusion of molecular complexes. *J. Chem. Phys.* **39**, 3042, doi:10.1063/1.1734140 (1963).
19 Breuer, K. H. & Eysel, W. The calorimetric calibration of differential scanning calorimetry cells. *Thermochim. Acta* **57**, 317, doi:10.1016/0040-6031(82)80043-9 (1982).
20 De Kruif, C. G., Van Miltenburg, J. C. & Blok, J. G. Molar heat capacities and vapor pressures of solid and liquid benzophenone. *J. Chem. Thermodyn.* **15**, 129 (1983).
21 Hanaya, M., Hikima, T., Hatase, M. & Oguni, M. Low-temperature adiabatic calorimetry of salol and benzophenone and microscopic observation of their crystallization: finding of homogeneous-nucleation-based crystallization. *J. Chem. Thermodyn.* **34**, 1173-1193, doi:10.1006/jcht.2002.0976 (2002).

22 Stejfa, V., Fulem, M., Ruzicka, K. & Moravek, P. New Static Apparatus for Vapor Pressure Measurements: Reconciled Thermophysical Data for Benzophenone. *J. Chem. Eng. Data* **61**, 3627-3639, doi:10.1021/acs.jced.6b00523 (2016).

23 Volmer, M. & Kirchhoff, P. Vapor pressure of solid and liquid benzophenone between 0° and 48°. *Z. physik. Chem.* **115**, 233 (1925).

24 Pribilova, J. & Pouchly, J. Vapor pressure of some low-volatility hydrocarbons determined by the effusion method. *Collect. Czech. Chem. Commun.* **39**, 1118, doi:10.1135/cccc19741118 (1974).

25 Colomina, M., Jimenez, P., Turrion, C., Fernandez, J. A. & Monzon, C. Vapor pressures and sublimation enthalpy of benzophenone. *An. Quim., Ser. A* **76**, 245 (1980).

26 Verevkin, S. P. Thermochemistry of aromatic ketones. Experimental enthalpies of formation and structural effects. *Thermochim. Acta* **310**, 229-235, doi:10.1016/s0040-6031(97)00231-1 (1998).

27 Monte, M. J. S., Santos, L. M. N. B. F., Fulem, M., Fonseca, J. M. S. & Sousa, C. A. D. New Static Apparatus and Vapor Pressure of Reference Materials: Naphthalene, Benzoic Acid, Benzophenone, and Ferrocene. *J. Chem. Eng. Data* **51**, 757-766, doi:10.1021/je050502y (2006).

28 De Kruif, C. G. & Oonk, H. A. J. The determination of enthalpies of sublimation by means of thermal conductivity manometers. *Chem.-Ing.-Tech.* **45**, 455, doi:10.1002/cite.330450705 (1973).

29 Van Ginkel, C. H. D., De Kruif, C. G. & De Waal, F. E. B. Need for temperature control in effusion experiments. *J. Phys. E* **8**, 490 (1975).

30 Sabbah, R. & Laffitte, M. Thermodynamic study of the benzophenone molecule. *Thermochim. Acta* **23**, 196 (1978).

31 Hulett, G. A. Der stetige Übergang fest-flüssig. *Ann. Phys. Chim.* **66**, 473-498 (1898).

32 Tammann, G. Über die Grenzen des festen Zustandes II. *Z.Phys.Chem.(Leipzig)* **28**, 629-672 (1899).

33 Deffet, L. Piezometric researches. I. Effect of high pressures on the temperatures of fusion and transformation of organic compounds. *Bull. Soc. Chim. Belg.* **44**, 97-139 (1935).

34 Akella, J. & Kennedy, G. C. Melting of three organic compounds at high pressures. *J. Chem. Phys.* **52**, 970, doi:10.1063/1.1673085 (1970).
